# Mechanical and Thermodynamic Properties of Non-Muscle Contractile Tissues: The Myofibroblast and the Molecular Motor Non-Muscle Myosin Type IIA

**DOI:** 10.3390/ijms22147738

**Published:** 2021-07-20

**Authors:** Yves Lecarpentier, Victor Claes, Jean-Louis Hébert, Olivier Schussler, Alexandre Vallée

**Affiliations:** 1 Centre de Recherche Clinique, Grand Hôpital de l’Est Francilien, 77100 Meaux, France; 2 Department of Pharmaceutical Sciences, University of Antwerp, 2180 Wilrijk, Belgium; victor.claes@scarlet.be; 3 Institut de Cardiologie, Hôpital de la Pitié-Salpêtrière, 75013 Paris, France; jean.l.hebert@gmail.com; 4 Département de Chirurgie Thoracique, Hôpital Cochin, Hôpitaux Universitaires Paris Centre, APHP, Paris-Descartes Université, 75014 Paris, France; olivier.schussler@gmail.com; 5 Department of Clinical Research and Innovation, Foch Hospital, 92150 Suresnes, France; alexandre.g.vallee@gmail.com

**Keywords:** myofibroblast, myosin, non-muscle myosin, TGF-β, mechanics, near equilibrium thermodynamics

## Abstract

Myofibroblasts are contractile cells found in multiple tissues. They are physiological cells as in the human placenta and can be obtained from bone marrow mesenchymal stem cells after differentiation by transforming growth factor-β (TGF-β). They are also found in the stroma of cancerous tissues and can be located in non-muscle contractile tissues. When stimulated by an electric current or after exposure to KCl, these tissues contract. They relax either by lowering the intracellular Ca^2+^ concentration (by means of isosorbide dinitrate or sildenafil) or by inhibiting actin-myosin interactions (by means of 2,3-butanedione monoxime or blebbistatin). Their shortening velocity and their developed tension are dramatically low compared to those of muscles. Like sarcomeric and smooth muscles, they obey Frank-Starling’s law and exhibit the Hill hyperbolic tension-velocity relationship. The molecular motor of the myofibroblast is the non-muscle myosin type IIA (NMIIA). Its essential characteristic is the extreme slowness of its molecular kinetics. In contrast, NMIIA develops a unitary force similar to that of muscle myosins. From a thermodynamic point of view, non-muscle contractile tissues containing NMIIA operate extremely close to equilibrium in a linear stationary mode.

## 1. Introduction

Up until now, when talking about contractile tissues, we have been referring to muscle tissues that include both sarcomeric skeletal and cardiac striated muscles and non sarcomeric smooth muscles. Their mechanical properties have been widely studied over the past century. More recently, the concept of non-muscle contractile tissues has emerged, referring to tissues that are clearly not muscles but which surprisingly exhibit contractile properties that share strong analogies with those of muscles themselves. What primarily distinguishes muscles from non-muscle tissues is their respective types of cells and molecular motors that generate specific contractile properties. In striated and smooth muscles, the basic contractile cell is the myocyte, and the molecular motors are the muscle myosin types I and II (MI and MII) [1]. In non-muscle tissues, the basic contractile cell is the myofibroblast, the molecular motor of which is the non-muscle myosin type IIA (NMIIA).

**Figure 1:**
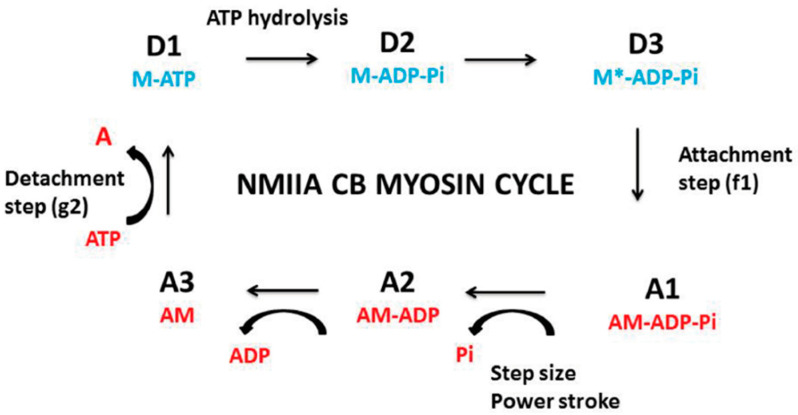
Crossbridge NMIIA cycle. The NMIIA CB cycle is composed of six main successive conformational steps, of which three are detached (D1, D2, and D3) and three are attached (A1, A2, and A3); (a) In transition A3 ⟹ D1: ATP binds with the actin- non-muscle myosin (AM) complex, which induces myosin (M) detachment from actin (**A**). AM + ATP ⟹ A + M-ATP; (b) In transition D1 ⟹ D2: the ATP hydrolysis step leads to M—ATP ⟹ M-ADP-Pi. (c) In transition D2 ⟹ D3: M-ADP-Pi ⟹ M*-ADP-Pi; (d) In transition D3 ⟹ A1: this leads to the attachment state: M*-ADP-Pi binds with A: M*-ADP-Pi + A ⟹ AM-ADP-Pi; (e) In transition A1 ⟹ A2: the power stroke is triggered by the release of Pi: AM-ADP-Pi ⟹ AM-ADP + Pi. The power stroke generates a unitary CB force and a swing of the myosin CB; (f) Transition A2 ⟹A3: release of ADP according to AM-ADP ⟹ AM + ADP.

A. Huxley’s formalism [2] provides a phenomenological tool to account for the behavior of CB molecular motors in both muscles and non-muscle contractile tissues. A. Huxley’s equations [2] can be used to determine mechanical properties of muscle myosins MI and MII and non-muscle myosins (NMII) at the molecular level, as well as the probabilities of occurrence of the different steps of the actin-myosin cycle (Figure 1). They make it possible to calculate total myosin content, maximum myosin ATPase activity, crossbridge (CB) rate constant of attachment and detachment, and the mean force developed by one CB. Due to the huge number of molecular motors per unit volume of contractile tissues, we can use statistical mechanics with the grand canonical ensemble to calculate numerous thermodynamic quantities such as statistical entropy, internal energy, chemical affinity, thermodynamic flow, thermodynamic force, and entropy production rate [3]. The grand canonical ensemble is a general method to apply statistical mechanics to the study of complex open systems such as contractile systems. Living open systems operate either near or far away from equilibrium [4,5]. Under physiological conditions, contractile tissues behave in a near-equilibrium manner and in a stationary linear regime [6,7,8]. We have devoted this review to both the mechanical and thermodynamic properties of non-muscle contractile tissues by comparing them to their muscle counterparts.

## 2. The Myofibroblast

The myofibroblast is the basic cell of several non-muscle contractile tissues. Myofibroblasts have been discovered by Gabbiani et al. during research on the presence of modified fibroblasts in the wound granulation tissue of healing skin [9]. Wound contracture, which accounts for the active retraction of the granulation tissue, is induced by activation of non-muscle contractile cells called myofibroblasts [10]. Myofibroblasts play a key role in numerous fibrotic diseases, such as idiopathic pulmonary fibrosis, systemic sclerosis, glomerular sclerosis, liver cirrhosis, or heart failure [11,12,13]. They are also involved in the stroma of epithelial cancers [14], human anterior capsular cataracts [15], and retinal detachment. During fibrosis, the contractile process is a retractile phenomenon associated with the synthesis of collagen in the extracellular matrix (ECM), leading to irreversible fibrosis and apoptosis of myofibroblasts.

Under physiological conditions, myofibroblasts are non-muscle contractile cells that are present in organs such as the stem villi of human placenta during normal pregnancies [16,17,18,19]. Importantly, they can alternately contract and relax, driving continuous changes in the volume of the intervillous chamber. In engineered tissues, human bone marrow-derived mesenchymal stem cells (MSCs) seeded in a collagen scaffold, MSCs in the presence of TGF-β1 differentiate into myofibroblasts and can contract and relax [20]. Myofibroblast differentiation is triggered by multiple cellular pathways [21,22]. TGF-β1 plays a key role in the differentiation of MSCs into myofibroblasts by upregulating the canonical Wnt/β-catenin signaling and downregulating PPARγ [12,13,23,24,25,26]. The Wnt/β-catenin pathway promotes fibrosis, whereas PPARγ prevents it. In numerous pathological states, these two pathways operate in an opposing manner [26]. In response to TGF-β1 stimulation, fibroblasts transdifferentiate into contractile myofibroblasts that express α-smooth muscle actin (α-SMA) and synthesize extracellular matrix (ECM) containing type I and type III collagen and ED-A fibronectin, which is essential for the myofibroblast differentiation [27]. The main ultrastructural properties of myofibroblasts are the presence of α-SMA, peripheral focal adhesions, and gap junctions [28]. TGF-β1 favors the synthesis of α-SMA, which leads to differentiation of fibroblasts into myofibroblasts. Incorporation of α-SMA into stress fibers significantly increases the contractile performance of myofibroblasts [29]. Differentiation into myofibroblasts can also occur through the process of epithelial–mesenchymal transition and endothelial–mesenchymal transition [30]. MSCs are myofibroblast precursors in numerous pathological states [12].

## 3. The Myofibroblast: A Contractile Cell Containing the Non-Muscle Myosin (NMIIA)

In non-muscle contractile cells, the molecular motor is the non-muscle myosin type II (NMII) [31]. There are three isoforms: NMIIA, NMIIB, and NMIIC. NMIIs are involved in generation of cell polarity, cell migration, and cell-cell adhesion. NMIIA is present in myofibroblasts located in the normal human placenta [18,19,32], in engineered tissues (from bone marrow MSC-seeded in collagen scaffold [20]), and in several pathological tissues such as cancers and fibrotic lesions (Dupuytren’s nodules, hypertrophic scars) [33].

## 4. The Non-Muscle Myosin (NMII)

Like muscle myosin, NMII contains three pairs of chains, i.e., two non-muscle heavy chains (NMHCs) of 230 kDa, two 20 kDa regulatory light chains (RLCs), and two 17 kDa essential light chains (ELCs). The structure of non-muscle myosin II (NMII) forms a dimer. Two globular motor domains (S-1) of the NMII contain binding sites for both the Mg^2+^-non-myosin ATPase and the actin-binding region. They are followed by neck regions, each of which binds two functionally different light chains, i.e., the ELCs and the RLCs that bind to the heavy chains at the level of the lever arms. The lever arms link the motor domains and rod domains. The neck domain acts as a lever arm to amplify the head rotation and is followed by a long α-helical coiled-coil that forms an extended rod-shaped domain and terminates in a short non-helical tail. In the absence of RLC phosphorylation, NMII forms a compact molecule through a head-to-tail interaction. This results in an assembly-incompetent form (10S). When RLC is phosphorylated, the 10S structure unfolds and leads to an assembly-competent form (6S). The heavy meromyosin (HMM) fragment contains the motor domain, the neck, and a part of the rod. This allows the dimerization of NMII molecular motors, which assemble into bipolar filaments via interactions between their rod domains. These filaments bind to actin through their head domains. The Mg^2+^-ATPase activity of the motor domain induces a conformational change that moves actin filaments in an anti-parallel manner. Bipolar myosin filaments link actin filaments together in thick bundles. This leads to cellular structures such as stress fibers. The ELCs are important stabilizers of the NMHC structure.

The NMII activity is regulated by two processes: firstly, by the calcium-calmodulin-myosin light chain kinase (MLCK); secondly, by the Rho/ROCK/myosin light chain phosphatase [34,35]. NMII binds with actin through the head domain of the heavy chain. Importantly, NMII molecules assembled into bipolar filaments allow the myosin molecules to slide along the actin filaments. A tilt of the motor domain enables a conformational change that moves actin filaments in an anti-parallel manner. The crossbridge (CB) actin-myosin cycle of NMII resembles that observed in smooth and striated muscle myosins (Figure 1).

The ATP molecule binds the NMII-ATPase site located on the motor domain. This allows the dissociation of actin from the NMII head. ATP is then hydrolyzed and subsequently, NMII binds with actin. Then, the power stroke occurs with a tilt of the NMII head, which generates a CB single force (Table 1) and a displacement of a few nanometers. ADP is then released from the actin-NMII complex. A new ATP molecule dissociates actin from the motor domain, and a new CB cycle begins.

## 5. Mechanical and Thermodynamic Techniques for Studying Contractile Tissues

### 5.1. Numerous Techniques

Numerous techniques have been used to measure force generated by fibroblasts and myofibroblasts on cell population or single cells embedded in a collagen lattice [35,36]. Contractile forces can be either indirectly measured by changes in the collagen scaffold volume or area, or directly measured by means of culture force monitors. Traction forces generated by individual myofibroblasts can also be measured by means of micropost force sensor array, cell traction force microscopy, wrinkle-able silicone membrane, and micro-machined cantilever beam array. In our laboratory, we use an electronic force transducer or micro-electronic force transducer to measure instantaneous force and shortening length of a contractile sample at all load levels from zero load up to isometric tension. A diffractometer can be added to measure sarcomere length in the case of striated muscles [37]. 

### 5.2. Experimental Set-Up

**Figure 2:**
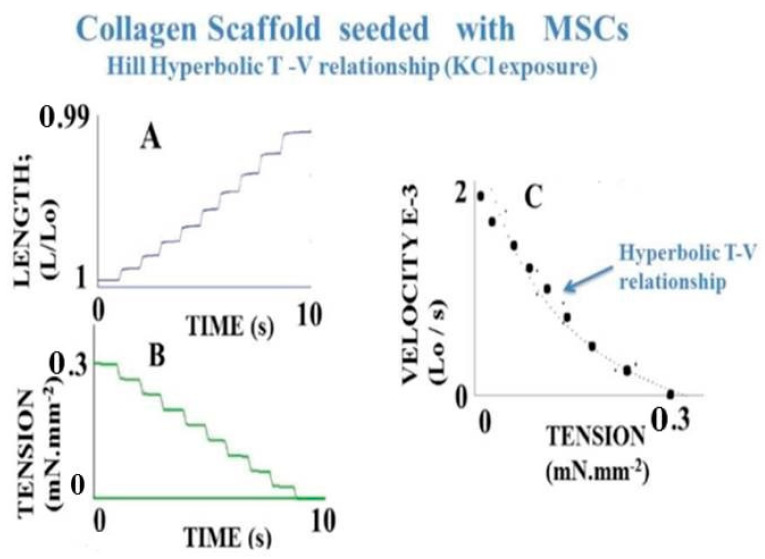
Hill hyperbolic tension (T)-velocity (V) relationship: The T-V hyperbolic relationship is established from a KCl-activated scaffold seeded with MSCs. Length (panel **A**) is progressively increased and tension (panel **B**) is progressively decreased by imposing several successive load clamps (LC). LC induces a brief transient overshoot. Maximum shortening velocity corresponding to a new isotonic load level is measured. (Panel **C**) represents the Hill hyperbolic T-V relationship. The curvature of the hyperbolic T-V relationship is introduced into Huxley’s equations to determine the molecular myosin properties (Table 1). This cardinal mechanical property is shared by both contractile muscles and non-muscle tissues.

Muscle and non-muscle contractile samples were carefully dissected and rapidly mounted in a chamber containing a Krebs-Henseleit solution bubbled with 95% o_2_-5% CO_2_ to maintain at the pH at 7.4. Contractile samples were stimulated either electrically or chemically by KCl (0.05M). Maximum unloaded shortening velocity (Vmax, in Lo. s^−1^) was measured by the zero-load clamp technique [38]. Lo is the resting length. Maximum isometric tension (maximum force normalized per cross-sectional area: To, in mN.mm^−2^) was measured from the isometric contraction. The A.V. Hill hyperbolic tension-velocity relationship [39] was calculated from maximum velocity (V) of 8–10 isotonic afterload contractions versus the level of isotonic tension (T), and by successive load increments from zero-load up to isometric tension (To) [40]. The tension-velocity (T-V) relationship was fitted according to (T + a) (V + b) = [To + a] b, where -a and -b are the hyperbola asymptotes. The G curvature of the T-V relationship was equal to To/a = Vmax/b [39,41] (Figure 2).

### 5.3. A. Huxley Formalism

#### 5.3.1. A. Huxley’s Equations

The phenomenological formalism of A. Huxley [2] is used to calculate several molecular parameters of myosin CBs which also makes it possible to determine the probabilities of the different steps in the CB cycle (Figure 1). Contractile tissues must present the hyperbolic T-V relationship, because the -a and -b asymptotes and the curvature G of the T-V relationship are part of Huxley’s equations. Using this formalism, the rate of total energy release (E_Hux_) and isotonic tension (P_Hux_) are expressed as follows:

(1) $$E_{\mathrm{Hux}}=\;(N\;e)\;(h/2\;l)(f_{1}/(f_{1}\;+\;g_{1}))\{g_{1}\;+\;f_{1}\;(V/\Phi )[(1\;-\;\exp\;(-\;\Phi /V)]\}$$

(2) $$P_{\mathrm{Hux}}=\;N\;(w/l)\;(f_{1}\;/(f_{1}\;+\;g_{1}))\{1\;-\;(V/\Phi )[(1\;-\;\exp\;(-\;\Phi /V))](1\;+\;(1/2)((f_{1}\;+\;g_{1})/g_{2}{)}^{2}(V/\Phi )\}$$

where w is the peak mechanical work generated by one CB (w/e = 0.75) and e is the free energy required to split one ATP molecule. The standard free energy G°′_ATP_ is −60 kJ/mol, and e is equal to 10^−19^ J [42]. The swing of the myosin CB ranges from 0 to h. The step size h of the CB myosin corresponds to the distance of translocation of the actin filament after the tilt of the motor domain. f_1_ is the maximum value of the rate constant for CB attachment; g_1_ and g_2_ are the maximum values of the rate constants for CB detachment; f_1_ and g_1_ correspond to a swing of the motor domain from 0 to h; g_2_ corresponds to a tilt > h; N is the number of cycling CBs per mm^2^ of cross-sectional area at maximum isometric tension. The constant l is the distance between two successive actin sites with which a myosin CB can bind. According to the A. Huxley conditions (l >> h), h and l values are respectively 10 nm and 28.6 nm. The parameters po, kcat, G curvature, f_1_, g_1_, and g_2_ are calculated using the following relationships:

G = f_1_/g_1_ (3)

g_1_ = 2wb/ehG (4)

g_2_ = 2Vmax/h

kcat = (h/2 l) × [(f_1_g_1_)/(f_1_ + g_1_)] (5)

po = (w/l) × [(f_1_)/(f_1_+g_1_)] (6)

where kcat is the catalytic constant (in s^−1^) and po is the single CB force (in pN). Myosin content is calculated from the number of cycling myosin CBs per mL of tissue and the Avogadro number. The maximum myosin ATPase activity (which is the thermodynamic flow) is the product of molar myosin concentration and kcat. At any given load level, the mechanical efficiency (Eff) of the contractile sample is calculated as the ratio of W_M_ to E_Hux_ and max.Eff is the peak value of efficiency (Table 1).

#### 5.3.2. Computation of CB Probabilities of the 6 States of the CB Cycle 

The myosin CB cycle of contractile tissues contains 6 main steps (Figure 1) of which 3 are detached (D1, D2, and D3) and 3 are attached (A1, A2, and A3). The probability of occurrence of a given step is calculated from the ratio of the duration of the step and the overall duration of the CB cycle tc = 1/kcat [43]. The probability PD1 is equal to the duration of step D1, i.e., tD1/tc = (1/g_2_)/tc = kcat/g_2_. The probability PA1 is equal to tA1/tc = (1/f_1_)/tc = kcat/f_1_. During the power stroke and step size h, the rate of mechanical energy is equal to eo = po × h. Probability PA2 is equal to h.po/e. The most probable detached state is D3, and the least probable state is A3. By convention, the lowest energy level (E_0_) coincides with the ground state E_0_ and is equal to zero: E_0_ = E_D3_ = 0 [3]. The ratio of probabilities of the most probable state and the least probable state is obtained from the relationship: E_A3_ − E_D3_ = kT (ln P_D3_/P_A3_) = e = 10 ^− 19^ J. The highest state level is E_A3_ = 10^−19^ J. Moreover, PA3 + PD3 = 1 − (PA1 + PA2 + PD1 + PD2). Because we know the ratio PD3/PA3 and the sum PA3 + PD3, we deduce PA3 and PD3.

### 5.4. Statistical Mechanics Near Equilibrium

Statistical mechanics (SM) near equilibrium can be applied to open contractile tissues. Living open contractile tissues exchange energy and matter with the surroundings and produce energy (ATP) that drives mechanical and thermodynamic processes. The grand canonical ensemble represents a general method for studying complex open systems. **S** is an open contractile system in a container **C**. The container **C** contains all the non-cycling myosin CBs and non-cycling actin molecules, and all the ATP, ADP, and Pi that are not attached to the cycling myosin CBs. Myosin CBs, actin molecules, and small soluble molecules (i.e., ATP, ADP, and inorganic phosphate Pi) can be exchanged between **S** and **C**. The system **S** is composed of all the active cycling myosin CBs individually found in a given state. In the grand canonical ensemble, the average number of independent, non-interacting cycling myosin CBs within **S** is determined from A. Huxley’s equations.

Let A be the chemical affinity of the CB cycle, S the statistical entropy, E the internal energy, and T (Kelvin) the temperature of the system. The grand potential (Ψ) is linked to S, A, E, and T according to the thermodynamic relationship Ψ = E − TS + A. Statistical entropy S is equal to the equation S = − R Σr Pr ln Pr.

The microcanonical partition function (z) is z = 1/x, (x being the highest probability PD3 of the CB cycle). The classic thermodynamic equation gives: E − T S = − RT ln z.

Thus, E − T S = Ψ − A = − RT ln z = RT ln x. The chemical affinity A is calculated from the equation A = Ψ − RT ln x.

The thermodynamic force is equal to A/T. When the affinity A is << RT (R: gas constant; T: Kelvin temperature, i.e., RT ≈ 2500 J/mol), a chemical system operates near-equilibrium. A near-equilibrium chemical system evolves towards a stationary state when the thermodynamic force (A/T) varies linearly with the thermodynamic flow [44,45]. The change in entropy dS is the sum of d_e_S and d_i_S, (d_i_S ≥ 0), in which d_e_S is the entropy change due to the exchange of matter and energy with the exterior, and d_i_S is the entropy change due to irreversible processes within the system. In linear stationary systems, the entropy production rate (d_i_S/dt) is the product of the thermodynamic force (A/T) and the thermodynamic flow [5,46]; d_i_S/dt can reach a minimum level that represents the criterion of stability of a stationary state. All the irreversible chemical processes are quantified by d_i_S/dt. The higher the value d_i_S/dt, the further the chemical system moves away from equilibrium.

## 6. Mechanical Properties Shared by Muscles and Non-Muscle Tissues 

**Figure 3:**
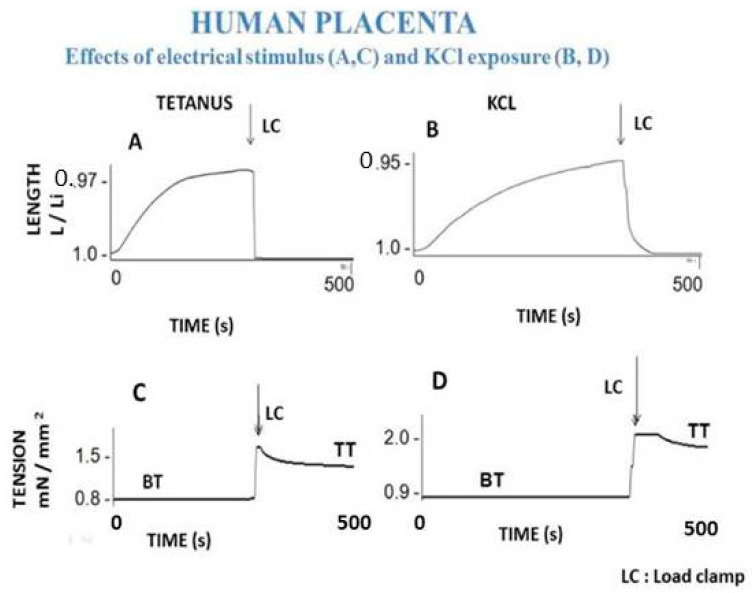
Basic contractility of human placental stem villi. (Panels **A** and **C**): Effect of tetanic electrical stimulation on placental stem villi. (Panel **A**): placental villi shortening. (Panel **C**): tension. When the shortening of the placental stem villi reaches a plateau, its value is at its maximum. The arrow marks the time at which tension is suddenly increased by means of a load clamp, placing the placental villi under isometric conditions. Active tension is the difference between total isometric tension and resting passive basal tension (BT). (Panels **B** and **D**): Effect of 0.05 M KCl on placental villi. (Panel **B**): placental stem villi shortening length. (Panel **D**): tension.

**Figure 4:**
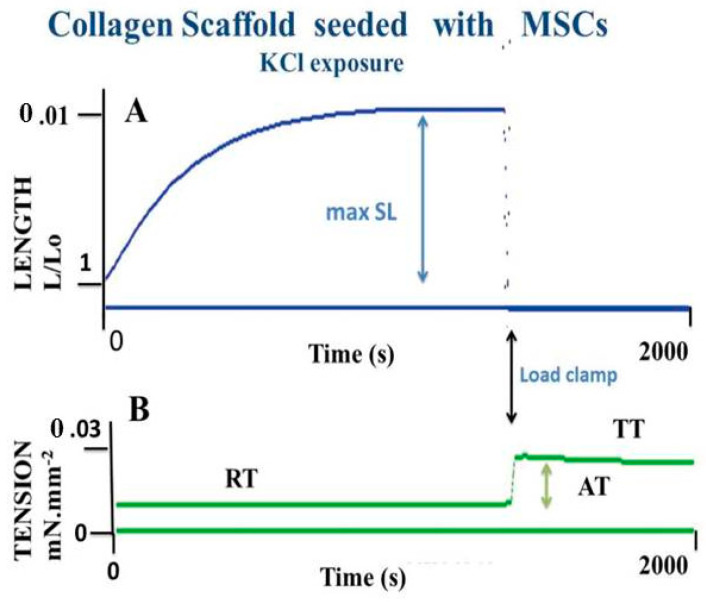
Basic contractility of an MSC-seeded collagen scaffold. Effect of 0.05 M KCl on a MSC-laden collagen scaffold. (Panel **A**): scaffold shortening. (Panel **B**): tension generation. When the collagen scaffold reaches a plateau, shortening length reaches a maximum (maxSL). The black arrow marks the time at which tension is suddenly increased, placing the collagen scaffold under isometric conditions. Active tension (AT) is the difference between total isometric tension (TT) and resting passive tension (RT).

**Figure 5:**
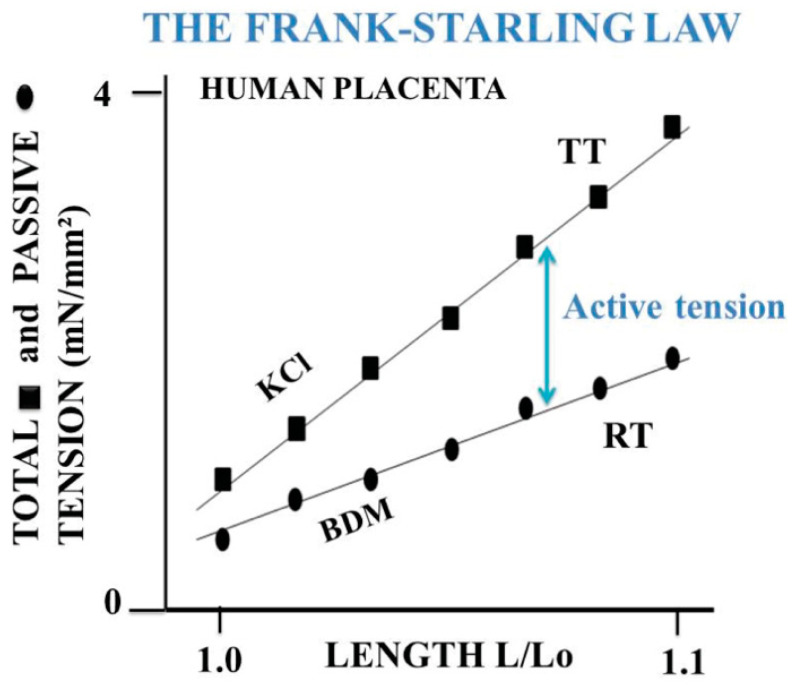
Frank-Starling mechanism. The Frank-Starling law indicates that when the initial length at rest (Lo) of the contractile sample increases, the active isometric tension also increases. The passive resting tension is measured in the presence of BDM, which inhibits the CB molecular motor interactions. Total isometric tension (TT in mN) is measured as a function of increasing initial length (Lo) of placental stem villi. Active isometric tension is the difference between total isometric tension and passive resting tension (RT) and increases when Lo increases. This cardinal mechanical property is shared by both contractile muscles and non-muscle contractile tissues.

**Figure 6:**
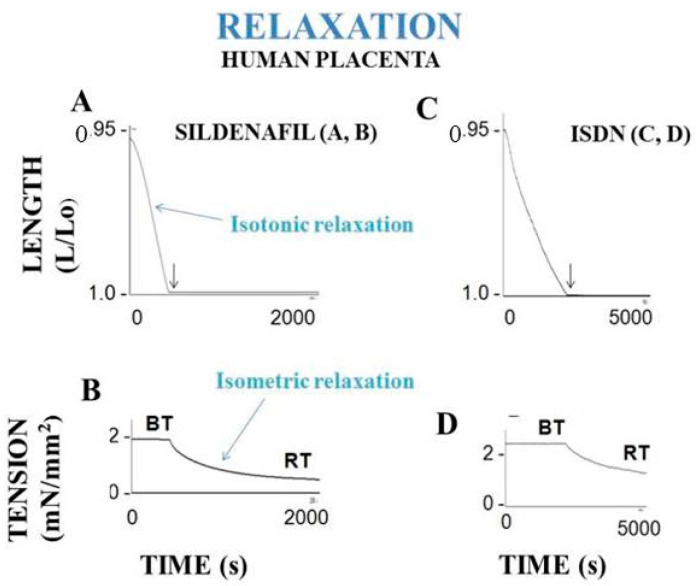
Relaxation of human placental stem villi. When maximum shortening length is reached, relaxation is obtained by either decreasing the intracellular Ca^2+^ concentration (by addition of Sildenafil or ISDN in the bath) or by inhibition of myosin CB (by BDM or BLE). Relaxing effect of Sildenafil is shown on (panels **A** and **B**). Relaxing effect of ISDN is shown on (panels **C** and **D**). Isotonic relaxation is followed by isometric relaxation.

Muscle and non-muscle contractile tissues share four main mechanical properties: (i) they contract after electrical stimulation (under either tetanic or twitch modes) or after KCl exposure (Figure 3 and Figure 4); (ii) they obey the Frank-Starling law, i.e., the developed tension increases when the initial length of the contractile sample increases [47,48] (Figure 5); (iii) they show a hyperbolic relationship between isotonic tension level (T) and peak shortening velocity (V) [39] (Figure 2) (importantly, the curvature of the T-V relationship can be introduced into A. Huxley’s equations for determining molecular characteristics of myosin CB (Table 1)); and (iv) they relax by decreasing the intracellular Ca^2+^ concentration (by means of isosorbide dinitrate (ISDN) or Sildenafil) or by inhibiting actin-myosin interaction (by means of either 2,3-butanedione monoxime (BDM) or blebbistatin (BLE) (Figure 6).

## 7. Mechanics of Two Non-Muscle Contractile Tissues: A Physiological Tissue (Human Placenta) and an Engineered Tissue (MSC-Seeded in a Collagen Scaffold)

### 7.1. Human Placenta

The human placenta is the prototype of non-muscle contractile physiological tissue, and it has long been suggested that it presents contractile properties [49]. Human placental stem villi (PSVs) contract parallel to their longitudinal axis. The contractile cells of the extravascular PSV stroma are arranged parallel to the longitudinal axis of the villi, unlike the circular vascular smooth muscle cells [18,50,51,52,53]. Smooth muscle-like cells have been described [54,55,56,57] in the extravascular part of human placental stem villi (PSVs) [50,58,59]. Moreover, maximum myosin ATPase activity and myosin content [60,61,62] both support the argument in favor of the human placenta presenting contractile properties. 

The mechanical contractile properties of human placenta were first described by Krantz and Parker [16], who observed the contraction of human PSVs when stimulated by KCl exposure. These results have been corroborated by Farley et al., who reported contraction and relaxation of PSVs [17] (Figure 3 and Figure 6). In isolated human PSVs, contraction can also be induced by electrical tetanus [19] (Figure 3A,C), whereas relaxation is induced by pharmacological agents inhibiting CB myosin (BDM and BLE) and by activating the NO-cGMP pathway (SIL and ISDN) (Figure 6).

Human PSV kinetics of contraction and relaxation are ultraslow, considerably slower than those observed in muscles [6,63] (Table 1). Importantly, the presence of myofibroblasts in human PSVs has been described by Feller et al. [18]. Extravascular cells of human PSVs express dipeptidyl peptidase IV, characteristic of myofibroblasts [18,59]. Moreover, NMIIA largely predominates in the PSV extravascular stromal tissue whereas the smooth muscle myosin type predominates in the vascular part of PSVs [32]. The dramatically slow maximum shortening velocity is partly accounted for by the very low maximum placental myosin ATPase activity (or thermodynamic flow) [60,62,64] (Table 1). This is corroborated by the very slow molecular kinetics of the non-muscle myosin NMIIA [65]. The low isometric tension reported in PSVs [16,17,19] is partly explained by the low placental myosin content [60,61,62] (Table 1). Human placenta villi obey the Frank-Starling law (Figure 5). The difference between total isometric tension and passive resting tension (i.e., active tension) increases as the PSV initial length increases. This mechanical property is the equivalent of the Frank-Starling mechanism observed in sarcomeric skeletal and cardiac muscles and in smooth muscle [66]. Relaxation of PSVs (Figure 6) is induced by inhibition of the actin-myosin CB (by means of BDM and BLE) or by stimulation of the NO-cGMP pathway. Activation of the NO-cGMP pathway leads to a decrease in [Ca^2+^]i in response to either ISDN or SIL. Huxley’s equations show the low value of maximum ATPase activity [60,62] (Table 1). The use of the Huxley formalism is possible due to the Hill hyperbolic T-V relationship (Figure 2). The single force generated by one CB does not significantly differ between muscle and non-muscle tissues (Table 1). Statistical mechanics shows the values of statistical entropy, affinity, thermodynamic force, and the extremely low values of both the thermodynamic flow and the entropy production rate when they are compared with those observed in the heart. PSVs operate near-equilibrium and in a stationary linear state (Table 1).

### 7.2. An Engineered Tissue: MSCs Seeded in Collagen Scaffolds

MSCs that reside in bone marrow (BM) can be amplified in vitro. BM-derived MSCs cultured in collagen scaffolds in the presence of TGF-β spontaneously differentiate into myofibroblasts exhibiting contractile properties. MSCs cultured in 2D form an adherent stroma of cells expressing well-organized microfilaments containing α-SMA and non-muscle myosin NMIIA. MSCs can be grown in 3D collagen scaffolds, generating a structure that develops contractile properties following exposure to KCl or stimulation by means of an electrical field [20]. Basic mechanical properties of collagen scaffolds seeded with MSCs, the molecular performance of NMII, and the mechanical statistics have been found to be quite similar to those observed in human PSVs [67,68] (Figure 2, Figure 3, Figure 4, Figure 5 and Figure 6) and Table 1). 

Like human placenta, collagen scaffolds seeded with MSCs operate near-equilibrium in a stationary linear state. NMIIA molecular kinetics are dramatically low in non-muscle myofibroblasts compared with values reported in cardiomyocytes [43]. However, CB unitary force is of the same order of magnitude in both myofibroblasts and cardiomyocytes (Table 1).

## 8. Synthesis

Non-muscle contractile tissues share major mechanical properties with muscles, but quantitatively, they differ considerably when compared to those observed in the heart (Table 1).

1. Their maximum shortening velocity (Vmax) is very slow due to their rate constant of detachment (g_2_) which is very low compared to that of the heart (due to Vmax proportional to g_2;_ see Equation (4)).
2. Their total isometric tension is very low due to their low myosin content compared to that of the heart.
3. The duration of their actin-myosin CB cycle (inverse of kcat) is very long due to their dramatically low CB attachment and detachment rate constants.
4. The elementary force developed by a single CB and the thermodynamic force are approximately of the same order of magnitude in all three contractile systems described in this study.
5. The thermodynamic flow and consequently the rate of entropy production (d_i_s) are dramatically low compared to those of the heart; d_i_s is the entropy change due to irreversible processes within the contractile system and is much lower in non-muscle contractile tissues than in the heart.
6. The 3 contractile tissues described here operate near-equilibrium and in a stationary linear regime. However, non-muscle contractile tissues (human placenta and MSC-seeded collagen scaffolds) behave in a manner that is nearer to equilibrium than that observed for the heart.

## 9. Conclusions

The NMIIA molecule is physiologically present in the human placenta and in bioengineered tissues in sufficient number to generate significant contractile properties. These non-muscle contractile tissues exhibit basic mechanical and thermodynamic properties similar to those observed in striated or smooth muscles. However, the values of their mechanical parameters are extremely low compared with those of muscles, as are their molecular myosin kinetics and most of their thermodynamic quantities. Moreover, the unitary force generated by a single NMIIA molecule is comparable to that generated by muscle myosins. It would be interesting to study the mechanics and thermodynamics of certain tissues that contain NMII, such as in the stroma of cancerous tissues.

## Figures and Tables

**Table 1:** Mechanical and thermodynamic properties of human placental stem villi, MSC-seeded in collagen scaffold, and heart muscle. Main mechanical parameters, molecular myosin characteristics, and thermodynamic quantities in human placental stem villi, MSCs seeded in collagen scaffold, and heart muscle. Only mean values are represented without SD.

	Placenta	MSC-Seeded Scaffold	Heart
Vmax (Lo/s)	0.002	0.002	3.7
Tension (mN)	1.5	0.3	42
kcat (s^−1^)	0.003	0.004	20.6
Unitary CB force (pN)	2.0	2.1	1.6
max Efficiency (%)	36	38	28
Myosin content (nmol/g)	0.14	0.07	12.5
G	3.7	4.1	1.6
PD3 (highest CB probability)	0.80	0.82	0.58
f1 (s^−1^)	0.08	0.08	306
g1 (s^−1^)	0.03	0.03	194
g2 (s^−1^)	0.34	0.35	731
Microcanonical partition function	1.25	1.22	1.72
Statistical entropy (J/T/mol)	5.6	10	9.3
Internal energy (J/mol)	1097	2000	1401
Affinity (J/mol)	354	250	535
Thermodynamic force	1.2	0.9	1.8
Thermodynamic flow (mol/L/s)	4.8 × 10^−13^	1.6 × 10^−13^	2.8
Entropy production rate (J/T/L/s)	8.2 × 10^−13^	1.8 x 10^−13^	6.5 × 10 ^−7^

## Data Availability

Not applicable.

## References

[1] Cooke R. (1997). Actomyosin interaction in striated muscle. Physiol. Rev..

[2] Huxley A.F. (1957). Muscle structure and theories of contraction. Prog. Biophys. Biophys. Chem..

[3] Atkins P.W. (1990). Physical Chemistry.

[4] Prigogine I. (1967). Introduction to Thermodynamics of Irreversible Processes.

[5] Kondepudi D., Prigogine I. (1999). Modern Thermodynamics from Heat Engines to Dissipative Structures.

[6] Lecarpentier Y., Claes V., Lecarpentier E., Blanc F.X., Joseph T., Geraets B., Krokidis X., Hebert J.L. (2011). Comparative statistical mechanics of myosin molecular motors in rat heart, diaphragm and tracheal smooth muscle. C. R. Biol..

[7] Lecarpentier Y., Blanc F.X., Quillard J., Hebert J.L., Krokidis X., Coirault C. (2005). Statistical mechanics of myosin molecular motors in skeletal muscles. J. Theor. Biol..

[8] Lecarpentier Y., Claes V., Hebert J.L., Krokidis X., Blanc F.X., Michel F., Timbely O. (2015). Statistical Mechanics of the Human Placenta: A Stationary State of a Near-Equilibrium System in a Linear Regime. PLoS ONE.

[9] Gabbiani G., Ryan G.B., Majne G. (1971). Presence of modified fibroblasts in granulation tissue and their possible role in wound contraction. Experientia.

[10] Gabbiani G., Hirschel B.J., Ryan G.B., Statkov P.R., Majno G. (1972). Granulation tissue as a contractile organ. A study of structure and function. J. Exp. Med..

[11] Hinz B. (2016). Myofibroblasts. Exp. Eye Res..

[12] Lecarpentier Y., Schussler O., Claes V., Vallée A. (2017). The Myofibroblast: TGFβ-1, A Conductor which Plays a Key Role in Fibrosis by Regulating the Balance between PPARγ and the Canonical WNT Pathway. Nucl. Recept. Res..

[13] Vallee A., Lecarpentier Y. (2019). TGF-beta in fibrosis by acting as a conductor for contractile properties of myofibroblasts. Cell Biosci..

[14] Hinz B., Phan S.H., Thannickal V.J., Prunotto M., Desmouliere A., Varga J., De Wever O., Mareel M., Gabbiani G. (2012). Recent developments in myofibroblast biology: Paradigms for connective tissue remodeling. Am. J. Pathol..

[15] Schmitt-Graff A., Pau H., Spahr R., Piper H.M., Skalli O., Gabbiani G. (1990). Appearance of alpha-smooth muscle actin in human eye lens cells of anterior capsular cataract and in cultured bovine lens-forming cells. Differ. Res. Biol. Div..

[16] Krantz E.K., Parker J.C. (1963). Contractile properties of the smooth muscle in the human placenta. Clin. Obstet. Gynecol..

[17] Farley A.E., Graham C.H., Smith G.N. (2004). Contractile properties of human placental anchoring villi. Am. J. Physiol. Regulat. Integrat. Comp. Physiol..

[18] Feller A.C., Schneider H., Schmidt D., Parwaresch M.R. (1985). Myofibroblast as a major cellular constituent of villous stroma in human placenta. Placenta.

[19] Lecarpentier E., Claes V., Timbely O., Hebert J.L., Arsalane A., Moumen A., Guerin C., Guizard M., Michel F., Lecarpentier Y. (2013). Role of both actin-myosin cross bridges and NO-cGMP pathway modulators in the contraction and relaxation of human placental stem villi. Placenta.

[20] Lecarpentier Y., Schussler O., Sakic A., Rincon-Garriz J.M., Soulie P., Bochaton-Piallat M.L., Kindler V. (2018). Human Bone Marrow Contains Mesenchymal Stromal Stem Cells That Differentiate In Vitro into Contractile Myofibroblasts Controlling T Lymphocyte Proliferation. Stem Cells Int..

[21] Leask A., Abraham D.J. (2004). TGF-beta signaling and the fibrotic response. FASEB J..

[22] Clevers H., Nusse R. (2012). Wnt/beta-catenin signaling and disease. Cell.

[23] Wei J., Fang F., Lam A.P., Sargent J.L., Hamburg E., Hinchcliff M.E., Gottardi C.J., Atit R., Whitfield M.L., Varga J. (2012). Wnt/beta-catenin signaling is hyperactivated in systemic sclerosis and induces Smad-dependent fibrotic responses in mesenchymal cells. Arthritis Rheum..

[24] Wei J., Ghosh A.K., Sargent J.L., Komura K., Wu M., Huang Q.Q., Jain M., Whitfield M.L., Feghali-Bostwick C., Varga J. (2010). PPARgamma downregulation by TGFss in fibroblast and impaired expression and function in systemic sclerosis: A novel mechanism for progressive fibrogenesis. PLoS ONE.

[25] Lakshmi S.P., Reddy A.T., Banno A., Reddy R.C. (2017). PPAR Agonists for the Prevention and Treatment of Lung Cancer. PPAR Res..

[26] Lecarpentier Y., Claes V., Duthoit G., Hebert J.L. (2014). Circadian rhythms, Wnt/beta-catenin pathway and PPAR alpha/gamma profiles in diseases with primary or secondary cardiac dysfunction. Front. Physiol..

[27] Serini G., Bochaton-Piallat M.L., Ropraz P., Geinoz A., Borsi L., Zardi L., Gabbiani G. (1998). The fibronectin domain ED-A is crucial for myofibroblastic phenotype induction by transforming growth factor-beta1. J. Cell Biol..

[28] Gabbiani G., Chaponnier C., Huttner I. (1978). Cytoplasmic filaments and gap junctions in epithelial cells and myofibroblasts during wound healing. J. Cell Biol..

[29] Hinz B., Celetta G., Tomasek J.J., Gabbiani G., Chaponnier C. (2001). Alpha-smooth muscle actin expression upregulates fibroblast contractile activity. Mol. Biol. Cell.

[30] Kim K.K., Kugler M.C., Wolters P.J., Robillard L., Galvez M.G., Brumwell A.N., Sheppard D., Chapman H.A. (2006). Alveolar epithelial cell mesenchymal transition develops in vivo during pulmonary fibrosis and is regulated by the extracellular matrix. Proc. Natl. Acad. Sci. USA.

[31] Conti M.A., Adelstein R.S. (2008). Nonmuscle myosin II moves in new directions. J. Cell Sci..

[32] Matsumura S., Sakurai K., Shinomiya T., Fujitani N., Key K., Ohashi M. (2011). Biochemical and immunohistochemical characterization of the isoforms of myosin and actin in human placenta. Placenta.

[33] Chiavegato A., Bochaton-Piallat M.L., D’Amore E., Sartore S., Gabbiani G. (1995). Expression of myosin heavy chain isoforms in mammary epithelial cells and in myofibroblasts from different fibrotic settings during neoplasia. Virchows Arch..

[34] Parizi M., Howard E.W., Tomasek J.J. (2000). Regulation of LPA-promoted myofibroblast contraction: Role of Rho, myosin light chain kinase, and myosin light chain phosphatase. Exp. Cell Res..

[35] Tomasek J.J., Gabbiani G., Hinz B., Chaponnier C., Brown R.A. (2002). Myofibroblasts and mechano-regulation of connective tissue remodelling. Nat. Rev. Mol. Cell Biol..

[36] Li B., Wang J.H. (2011). Fibroblasts and myofibroblasts in wound healing: Force generation and measurement. J. Tissue Viability.

[37] Lecarpentier Y., Martin J.L., Claes V., Chambaret J.P., Migus A., Antonetti A., Hatt P.Y. (1985). Real-time kinetics of sarcomere relaxation by laser diffraction. Circ. Res..

[38] Brutsaert D.L., Claes V.A., Goethals M.A. (1973). Effect of calcium on force-velocity-length relations of heart muscle of the cat. Circ. Res..

[39] Hill A.V. (1938). The heat of shortening and the dynamic constants of muscle. Proc. R. Soc. Lond. Biol. Sci..

[40] Lecarpentier Y., Chemla D., Blanc F.X., Pourny J.C., Joseph T., Riou B., Coirault C. (1998). Mechanics, energetics, and crossbridge kinetics of rabbit diaphragm during congestive heart failure. FASEB J..

[41] Woledge R.C., Curtin A.N., Homsher E. (1985). Energetic Aspects of Muscle Contraction.

[42] Veech R.L., Lawson J.W., Cornell N.W., Krebs H.A. (1979). Cytosolic phosphorylation potential. J. Biol. Chem..

[43] Lecarpentier Y., Claes V., Krokidis X., Vallée A.A. (2017). Comparative Statistical Mechanics of Muscle and Non-Muscle Contractile Systems: Stationary States of Near-Equilibrium Systems in A Linear Regime. Entropy J..

[44] Onsager L. (1931). Reciprocal relations in irreversible processes II. Phys. Rev..

[45] Prigogine I. (1986). Life and physics. New perspectives. Cell Biophys..

[46] De Donder T. (1927). L’ affinité.

[47] Frank O. (1895). Zur Dynamik des Herzmuskels. Z. Biol..

[48] Starling E.H. (1918). The Linacre Lecture on the Law of the Heart.

[49] Benirschke K., Kaufmann P., Baergen R.N. (2006). Pathology of the Human Placenta.

[50] Graf R., Langer J.U., Schonfelder G., Oney T., Hartel-Schenk S., Reutter W., Schmidt H.H. (1994). The extravascular contractile system in the human placenta. Morphological and immunocytochemical investigations. Anat. Embryol..

[51] Demir R., Kosanke G., Kohnen G., Kertschanska S., Kaufmann P. (1997). Classification of human placental stem villi: Review of structural and functional aspects. Microsc. Res. Tech..

[52] Sparn H.G., Lieder-Ochs B.A., Franke W.W. (1994). Immunohistochemical identification and characterization of a special type of desmin-producing stromal cells in human placenta and other fetal tissues. Differ. Res. Biol. Div..

[53] Kohnen G., Castellucci M., Hsi B.L., Yeh C.J., Kaufmann P. (1995). The monoclonal antibody GB 42—A useful marker for the differentiation of myofibroblasts. Cell Tissue Res..

[54] Iizuka S. (1916). Uber Verkammen von Muskelfasern in der menschlichen Placenta. Beitr. Geburtsh. Gynaek..

[55] Naujoks H. (1922). Heben anatomische Veränderungen der kindlichen Eihäute einen Einfluss auf die Zeit des Blasensprunges. Z. Geburtsh. Gynaek..

[56] Dubreuil G., Rivière M. (1932). Formations fibromusculaires du chorion et villosités du placenta humain. C. R. Soc. Biol..

[57] Spanner R. (1935). Mütterlicher und kindlicher kreislauf der menschlichen Placenta und seine Strömbahnen. Z. Anat. Entwicklungsgesch..

[58] Graf R., Schonfelder G., Muhlberger M., Gutsmann M. (1995). The perivascular contractile sheath of human placental stem villi: Its isolation and characterization. Placenta.

[59] Graf R., Matejevic D., Schuppan D., Neudeck H., Shakibaei M., Vetter K. (1997). Molecular anatomy of the perivascular sheath in human placental stem villi: The contractile apparatus and its association to the extracellular matrix. Cell Tissue Res..

[60] King T.M., Groeschel-Stewart U. (1965). Placental Contractile Protein. Am. J. Obstet. Gynecol..

[61] Michael C. (1974). Actomyosin content of the human placenta. J. Obstet. Gynaecol..

[62] Huszar G., Bailey P. (1979). Isolation and characterization of myosin in the human term placenta. Am. J. Obstet. Gynecol..

[63] Lecarpentier E.E., Claes V.A., Timbely O., Arsalane A., Wipff J.A., Hebert J.L., Michel F.Y., Lecarpentier Y.C. (2011). Mechanics and energetics of myosin molecular motors from non-pregnant human myometrium. J. Appl. Physiol..

[64] Lecarpentier Y., Claes V., Lecarpentier E., Guerin C., Hebert J.L., Arsalane A., Moumen A., Krokidis X., Michel F., Timbely O. (2014). Ultraslow myosin molecular motors of placental contractile stem villi in humans. PLoS ONE.

[65] Kovacs M., Wang F., Hu A., Zhang Y., Sellers J.R. (2003). Functional divergence of human cytoplasmic myosin II: Kinetic characterization of the non-muscle IIA isoform. J. Biol. Chem..

[66] Gordon A.M., Huxley A.F., Julian F.J. (1966). The variation in isometric tension with sarcomere length in vertebrate muscle fibres. J. Physiol..

[67] Lecarpentier Y., Kindler V., Bochaton-Piallat M.L., Sakic A., Claes V., Hebert J.L., Vallee A., Schussler O. (2019). Tripeptide Arg-Gly-Asp (RGD) modifies the molecular mechanical properties of the non-muscle myosin IIA in human bone marrow-derived myofibroblasts seeded in a collagen scaffold. PLoS ONE.

[68] Lecarpentier Y., Kindler V., Krokidis X., Bochaton-Piallat M.L., Claes V., Hebert J.L., Vallee A., Schussler O. (2020). Statistical Mechanics of Non-Muscle Myosin IIA in Human Bone Marrow-Derived Mesenchymal Stromal Cells Seeded in a Collagen Scaffold: A Thermodynamic Near-Equilibrium Linear System Modified by the Tripeptide Arg-Gly-Asp (RGD). Cells.

